# Elucidation of the mechanism behind the potentiating activity of baicalin against *Burkholderia cenocepacia* biofilms

**DOI:** 10.1371/journal.pone.0190533

**Published:** 2018-01-02

**Authors:** Lisa Slachmuylders, Heleen Van Acker, Gilles Brackman, Andrea Sass, Filip Van Nieuwerburgh, Tom Coenye

**Affiliations:** 1 Laboratory of Pharmaceutical Microbiology, Ghent University, Ghent, Belgium; 2 Laboratory of Pharmaceutical Biotechnology, Ghent University, Ghent, Belgium; University of Technology Sydney, AUSTRALIA

## Abstract

Reduced antimicrobial susceptibility due to resistance and tolerance has become a serious threat to human health. An approach to overcome this reduced susceptibility is the use of antibiotic adjuvants, also known as potentiators. These are compounds that have little or no antibacterial effect on their own but increase the susceptibility of bacterial cells towards antimicrobial agents. Baicalin hydrate, previously described as a quorum sensing inhibitor, is such a potentiator that increases the susceptibility of *Burkholderia cenocepacia* J2315 biofilms towards tobramycin. The goal of the present study is to elucidate the molecular mechanisms behind the potentiating activity of baicalin hydrate and related flavonoids. We first determined the effect of multiple flavonoids on susceptibility of *B*. *cenocepacia* J2315 towards tobramycin. Increased antibiotic susceptibility was most pronounced in combination with apigenin 7-O-glucoside and baicalin hydrate. For baicalin hydrate, also other *B*. *cepacia* complex strains and other antibiotics were tested. The potentiating effect was only observed for aminoglycosides and was both strain- and aminoglycoside-dependent. Subsequently, gene expression was compared between baicalin hydrate treated and untreated cells, in the presence and absence of tobramycin. This revealed that baicalin hydrate affected cellular respiration, resulting in increased reactive oxygen species production in the presence of tobramycin. We subsequently showed that baicalin hydrate has an impact on oxidative stress via several pathways including oxidative phosphorylation, glucarate metabolism and by modulating biosynthesis of putrescine. Furthermore, our data strongly suggest that the influence of baicalin hydrate on oxidative stress is unrelated to quorum sensing. Our data indicate that the potentiating effect of baicalin hydrate is due to modulating the oxidative stress response, which in turn leads to increased tobramycin-mediated killing.

## Introduction

*Burkholderia cepacia* complex (Bcc) bacteria are opportunistic pathogens, which cause severe lung infections in immunocompromised persons, such as cystic fibrosis (CF) patients [1]. The most frequently isolated Bcc species from these patients are *Burkholderia cenocepacia* and *Burkholderia multivorans* [2]. Infections with these pathogens are particularly difficult to treat due to their ability to form biofilms [3]. Biofilms are defined as communities of microbial cells embedded in a self-produced matrix that, compared to their planktonic counterparts, show reduced susceptibility towards antimicrobial therapy [4]. The process of biofilm formation is partially controlled by quorum sensing (QS), a cell-density-dependent communication system, that coordinates expression of various virulence factors [5,6]. *B*. *cenocepacia* has two acylhomoserine lactone (AHL) based systems, namely CepIR and CciIR. The CepIR system is present in all Bcc strains, while the CciIR system is only present in highly transmissible ET12 strains containing the cci genomic island. The CepIR system is generally responsible for positive regulation of QS-regulated genes while CciIR mainly acts as a negative regulator [7]. Another QS system in *B*. *cenocepacia* uses cis-2-dodecenoic acid, also referred to as BDSF (*Burkholderia* Diffusible Signal Factor) as signalling molecule. BDSF is synthesized by RpfF and sensed by RpfR [8]. There is a complex interplay between the AHL- and BDSF-based QS systems [9].

One of the mechanisms contributing to biofilm tolerance is the protection against oxidative stress [10]. These responses to oxidative stress are controlled by two major transcriptional regulators, OxyR and SoxRS [11], and include the production of polyamines, such as putrescine, which reduce intracellular reactive oxygen species (ROS) levels and protect membranes from lipid peroxidation [12,13].

It was previously described [14,15] that antibiotics also induce intracellular ROS production and it was shown that this also occurs in Bcc strains [16]. The primary drug-target interactions are thought to stimulate the oxidation of nicotinamide adenine dinucleotide (NADH) through the electron transport chain (ETC), which depends on the tricarboxylic acid cycle (TCA) [15,17]. Hyperactivation of the ETC leads to increased superoxide (O_2_^-^) production. These highly toxic ROS damage iron-sulphur clusters in proteins, making ferrous iron available for the Fenton reaction [15]. In this reaction, ferrous iron (Fe^2+^) will be oxidized by hydrogen peroxide (H_2_O_2_) to produce ferric iron (Fe^3+^) and deleterious hydroxyl radicals (^**·**^OH). ROS can directly damage macromolecules such as DNA, lipids and proteins [18] or indirectly damage DNA by oxidizing the deoxynucleotide pool [19].

A decreased activity of the tricarboxylic acid (TCA) cycle leads to a larger fraction of metabolically less active cells, in which endogenous ROS production is reduced [20]. This leads to increased tolerance towards antibiotics [21,22]. A lower activity of the TCA cycle is typically associated with an induction of the glyoxylate shunt. This shunt allows the cells to avoid NADH formation in the TCA cycle and thus avoid ROS production. This was already described for *P*. *aeruginosa* and *B*. *cenocepacia* strains exposed to lethal doses of aminoglycosides [11,16,20].

A promising approach to overcome tolerance and/or resistance is the use of antibiotic adjuvants, also described as potentiators. These are compounds with little or no intrinsic antibiotic activity that increase the susceptibility of bacterial cells towards antimicrobial therapy [23]. Brackman et al. [24] already demonstrated an increased susceptibility of *B*. *cenocepacia* biofilms towards tobramycin (TOB) when it was combined with the potentiator baicalin hydrate (BH). Baicalin (5,6-dihydroxy-7-O-glucuronide flavone), a flavonoid isolated from the roots of *Scutellaria baicalensis*, was described as an inhibitor of QS [24] and has a long history of use in Chinese medicine [25]. The goal of the present research is to elucidate the molecular mechanism behind the potentiating activity of BH and other flavonoids.

## Materials and methods

### Strains and culture conditions

The strains used in the present study are listed in Table 1. The strains were stored at -80°C using Microbank vials (Prolab Diagnostics, Richmond Hill, ON, Canada) and subcultured at 37°C on Trypton Soy agar (TSA; Lab M, Lancashire, UK) or TSA supplemented with 800 μg/ml trimethoprim (Ludeco, Brussels, Belgium) for MDL2. Overnight cultures were grown aerobically in Mueller Hinton broth (MHB; Lab M) at 37°C. Except for cultures on which the H_2_DCFDA assay was performed, Luria Bertoni agar (LBA; Lab M) and Luria Bertoni broth (LBB; Lab M) were used.

**Table 1:** Strains used in the present study.

Strain	Strain info	Source and/or reference
*B*. *cenocepacia* strains		
J2315 (LMG 16656^T^)	CF patient, UK, ET12 strain	BCCM/LMG bacteria collection (Ghent, University, Belgium)
Triple QS deletion mutant	J2315 *ΔcepIΔcciIΔrpfF*	G. Riccardi [26]
C5424 (LMG 18827)	CF patient, Canada, ET12 strain	BCCM/LMG bacteria collection
MDL2	C5424 *ΔkatB*	M. Valvano [27]
K56-2 (LMG 18863)	CF patient, Canada, ET12 strain	BCCM/LMG bacteria collection
OME11	K56-2 *Δ*BCAL2641	M. Valvano [28]
HI2424 (LMG 24507)	Soil, USA, PHDC strain	BCCM/LMG bacteria collection
AU1054 (LMG 24506)	CF patient, USA, PHDC strain	BCCM/LMG bacteria collection
C6433 (LMG 18828)	CF patient, Canada	BCCM/LMG bacteria collection
PC184 (LMG 18829)	CF patient, USA	BCCM/LMG bacteria collection
*B*. *multivorans* strains		
LMG 13010^T^	CF patient, Belgium	BCCM/LMG bacteria collection
LMG 18825	CF patient, UK	BCCM/LMG bacteria collection
*B*. *ambifaria* LMG 19182^T^	Pea rhizosphere, USA	BCCM/LMG bacteria collection

### Reagents

The following antibiotics were tested during the present study: tobramycin (TOB; TCI Europe, Zwijndrecht, Belgium), gentamicin (GN; Sigma-Aldrich), kanamycin (KN; Sigma-Aldrich), neomycin (NEO; Sigma-Aldrich), ceftazidime (CEF; Sigma-Aldrich), meropenem (MEM; Fresenius Kabi, Schelle, Belgium), minocycline (MIN; Sigma-Aldrich), ciprofloxacin (CIP; Sigma-Aldrich) and trimethoprim/sulfamethoxazole (Sigma-Aldrich) (co-trimoxazole, SXT). All antibiotics were dissolved in either MilliQ water (MQ water) (Millipore, Billerce, MA, US) to determine the minimal inhibitory concentration (MIC) or in physiological saline (PS) (0.9% w/v NaCl) (Applichem, Darmstadt, Germany) to treat biofilms. Stock solutions were filter sterilized (0.22 μm Whatman, Dassel, Germany) and stored at 4°C until use.

Structural derivatives of BH (Sigma-Aldrich, Bornem, Belgium) were selected to determine their potentiating activity in combination with TOB. These derivatives were scutellarin (Sigma-Aldrich), luteolin 7-O-glucoside (Sigma-Aldrich), schaftoside (Extrasynthese, Genay Cedex, France), myricitrin (Sigma-Aldrich) and apigenin 7-O-glucoside (Sigma-Aldrich). Stock solutions of the flavonoids were prepared in dimethyl sulfoxide (DMSO; Sigma-Aldrich) and diluted to a final solution of 1% with MQ water to determine the MIC or with PS to treat biofilms. A control with the same percentage of DMSO was included. A stock solution of sodium azide (NaN_3_) (Sigma-Aldrich) was prepared in MQ and further diluted in MHB prior to use.

### Determination of the minimal inhibitory concentration

MICs were determined according to the EUCAST broth microdilution assay using flat-bottom 96-well microtiter plates (MTP; SPL Lifescience, Korea) [29]. The flavonoid concentrations ranged from 4 μM to 500 μM. CEF, MEM, MIN and CIP concentrations tested ranged from 0.5 μg/ml to 512 μg/ml; SXT concentrations tested ranged from 0.25/4 μg/ml to 256/4864 μg/ml. All aminoglycosides (TOB, GN, KN and NEO) were tested in a concentration range from 0.5 μg/ml to 4096 μg/ml. The MIC was defined as the lowest concentration with a similar optical density as un-inoculated growth medium. Absorbance was measured at 590 nm with a multilabel MTP reader (EnVision, Perkin Elmer LAS, Waltham, MA). All MIC determinations were performed in triplicate.

### Biofilm formation

Biofilms were grown in clear round-bottomed 96-well plates (SPL) to evaluate their survival after treatment, or in black flat-bottomed 96-well plates (Perkin Elmer) for measuring fluorescence. An inoculum of approximately 5x10^7^ CFU/ml was prepared in fresh medium from an overnight culture. 100 μl of this inoculum was added to the wells of a MTP. After 4 hours of adhesion the supernatant was removed and the wells were rinsed with PS. Subsequently, fresh medium was added to the wells and the MTP was further incubated for 20 hours at 37°C.

### Biofilm treatment

To evaluate the effect of flavonoids on the susceptibility of biofilms towards antibiotics, biofilms were treated with following components: the antibiotic alone, the flavonoid alone, a combination of both, or PS as a control. All antibiotics were tested at concentrations of 4xMIC. The concentration of flavonoids was 100 μM to initially detect their potentiating activity. In subsequent experiments, a concentration of 250 μM was used for BH. All solutions were diluted in PS. When a stock solution was prepared in DMSO, a control with the same percentage DMSO was included. Biofilms were grown as described above. After 24 hours of biofilm formation the supernatant was removed and the wells were rinsed with PS. Subsequently, PS (= control), the antibiotic alone, the flavonoid alone or a combination of both was added to the wells. After 24 hours at 37°C, the supernatant was removed and the wells were rinsed with PS. Sessile cells were harvested from the MTP by two cycles of shaking (5 min, 900 rpm; Titramax 1000, Heidolph Instruments, Schwabach, Germany) and sonicating (5 min; Branson 3510, Branson Ultrasonics Corp, Danbury, CT, USA). The number of surviving cells (CFU/ml) was determined by plating the resulting bacterial suspension.

### Transcriptomic analysis

To elucidate the molecular mechanism by which BH affects biofilm susceptibility towards TOB, transcriptomes of treated and untreated *B*. *cenocepacia* J2315 biofilm cells were compared using RNA sequencing. Gene expression was determined in 24 hour-old *B*. *cenocepacia* J2315 biofilms that were exposed to TOB alone (3 x MIC), BH alone (250 μM), a combination of both, or PS (= control) for 24 hours. These concentrations were selected because sufficient living cells remained for the RNA extraction, while a significant difference between TOB and TOB+BH could be observed. For each treatment, three biological replicates were included. Biofilm cells were harvested as described above with two cycles of vortexing and sonicating. Total RNA was extracted using Ambion RiboPure Bacteria Kit (Ambion, Austin, TX) according to the manufacturers’ instructions, including DNAse treatment for 1 hour at 37°C. The concentration and quality of the total extracted RNA was determined by using the Quant-it ribogreen RNA assay (Life Technologies, Grand Island, NY, USA) and the RNA 6000 pico chip (Agilent Technologies, Santa Clara, CA, USA), respectively. Subsequently, 200 ng of RNA was depleted for rRNA using the Ribo-Zero Magnetic Kit for Gram-negative Bacteria (Epicentre, Madison, WI, USA). Library preparation was performed using the Truseq stranded Total RNA library prep (Illumina, San Diego, CA, USA) according to manufacturer's instructions. Libraries were quantified by qPCR, according to Illumina's Sequencing Library qPCR Quantification protocol guide, version February 2011. A DNA 1000 chip (Agilent Technologies, Santa Clara, CA, US) was used to verify the library's size distribution and quality. Sequencing was performed on a high throughput Illumina NextSeq 500 flow cell generating 75 bp single reads. After an initial quality control using CLC Genomics Workbench version 8.5.1 (Qiagen, Venlo, Netherlands), the reads for each condition were mapped to the reference genome sequences (accession numbers AM747720, AM747721, AM747722, and AM747723) [30] (Cut-offs: 90% length and 80% similarity). The number of reads per transcript were divided by the transcript length and then normalized to the total amount of reads, obtaining reads per kb per million (RPKM) expression values. Statistical analysis was performed using Empirical DGE test in CLC genomics Workbench version 8.5.1. The effect of the addition of BH to treated cells (TOB) or untreated cells (PS) on gene expression was evaluated. The combination of TOB+BH was compared to treatment with TOB alone, and treatment with BH alone was compared to an untreated control (PS) to analyse the effect of BH on both treated and untreated cells. Only genes that were significantly differentially expressed (p-value < 0.05) and with at least a 1.5-fold change were considered. Results were evaluated using the KEGG Pathway Database [31] and *Burkholderia* Genome Database [32]. The experimental protocols and the raw sequencing data can be found in ArrayExpress under the accession number E-MTAB-6099.

### Fluorometric determination of reactive oxygen species

To evaluate endogenous ROS production, a 2',7'-dichlorodihydrofluorescein diacetate (H_2_DCFDA)-based assay was used. H_2_DCFDA is a colourless, non-fluorescent compound that passively diffuses into the cell, where non-specific intracellular esterases cleave the acetate groups and so trap the compound in the cell. The cleaved product will be easily oxidized by intracellular ROS, yielding highly fluorescent 2',7'-dichlorofluorescein (DCF) [20]. Cells were pre-incubated with the dye before treatment to exclude differences in fluorescence due to an altered uptake by treated cells. Since this assay has been described as highly pH dependent, a pH-matched control was included [16]. For this assay, biofilms were cultivated as described above, while planktonic cultures were grown aerobically for 24 hours and were standardized to an optical density of 1 (λ = 590 nm). Biofilms and planktonic cultures were grown in LBB. After 24 hours the cells were rinsed with PS and incubated with 10 μM H_2_DCFDA in LBB shielded from light at 37°C. After 45 minutes the cells were rinsed with phosphate buffered saline (PBS) and treated with TOB (4xMIC), BH (250 μM) or a combination of both. A pH-matched control in PBS was included as a control for each condition. Fluorescence (λ excitation = 485 nm, λ emission = 535 nm) was measured using an Envision multilabel MTP reader. Net fluorescence was calculated by subtracting autofluorescence of bacterial cells incubated under the same conditions without H_2_DCFDA. Each experiment included at least three biological replicates.

### Statistical data analysis

Statistical analysis was performed using SPSS version 24 software (SPSS, Chicago, IL, USA). The Shapiro-Wilk test was used to verify the normal distribution of the data. Normally distributed data were analysed using an ANOVA or an independent sample T-test. Non-normally distributed data were analysed using a Kruskal-Wallis test or a Mann-Whitney test. P-values smaller than 0.05 were considered significant.

## Results and discussion

### Determination of the potentiating effect of flavonoids on the antibiotic susceptibility of Bcc species

**Fig 1:**
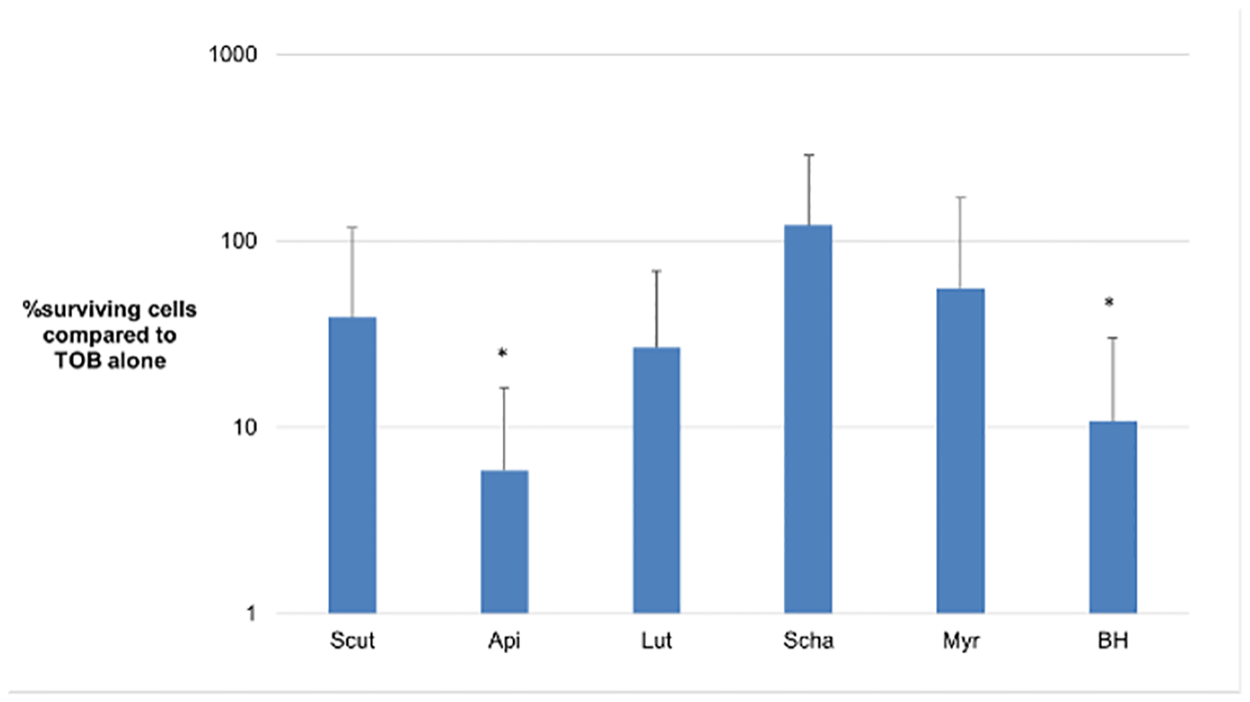
Potentiating effect of BH and other flavonoids. Data shown are percentage survival of *B*. *cenocepacia* J2315 biofilm cells treated with the combination of a flavonoid (100 μM) and TOB (4 x MIC) compared to TOB alone. The tested flavonoids were scutellarin (scut), apigenin 7-O-glucoside (api), luteolin 7-O-glucoside (lut), schaftoside (scha), myricitrin (myr) and baicalin hydrate (BH). *: statistically significant (p < 0.05) less survival compared to TOB alone. Error bars are standard deviations (SD) (n = 4).

The ability of several structural analogues of BH to increase the susceptibility of *B*. *cenocepacia* J2315 towards TOB was evaluated. The analogues tested were scutellarin, luteolin 7-O-glucoside, schaftoside, myricitrin and apigenin 7-O-glucoside. First, the MIC on *B*. *cenocepacia* was determined in order to select a flavonoid concentration which did not inhibit growth of the bacterial cells (sub-MIC). For all flavonoids the MIC values were >500 μM for *B*. *cenocepacia* J2315. To limit the amount of DMSO in the final solution to 1%, a concentration of 100 μM was selected. When biofilms were treated with BH or apigenin 7-O-glucoside, an increased killing was observed compared to treatment with TOB alone (Fig 1). No potentiating effect was observed with any of the other flavonoids tested. These results were not surprising since small structural differences in flavonoids can influence their antimicrobial activity [33,34].

The effect of BH in combination with TOB on the susceptibility of *B*. *cenocepacia* J2315 was already established [24]. This raised the question if BH could increase the susceptibility of *B*. *cenocepacia* J2315 biofilms towards other antibiotics. Therefore, several antibiotics (CEF, CIP, MIN, MEM and SXT) belonging to different classes were tested in combination with BH. However, the addition of BH did not lead to a significantly increased susceptibility towards any of the antibiotics tested (S1 Fig and S1 Table). This suggests that the increased susceptibility towards TOB is specific to aminoglycosides. To test this hypothesis, other aminoglycosides (GN, KN and NEO) were tested in combination with BH against *B*. *cenocepacia* J2315 and other Bcc strains (Table 1). Since BH has no antibacterial effect, a significant antibacterial effect of the antibiotic alone is required in order to observe the potentiating influence of BH. Therefore, strains with a high innate resistance towards aminoglycosides (MIC ≥ 1024 μg/ml) were not included (S2 Table). The reduction in surviving cells after treatment with the combination compared to the aminoglycoside alone is shown in Table 2.

**Table 2:** Potentiating effect of BH in Bcc biofilms.

Strain	TOB+BH vs. TOB	GN+BH vs. GN	KN+BH vs. KN	NEO+BH vs. NEO
*B*. *cenocepacia* J2315^T^	88.9 (± 10.3)	80.6 (± 14.6)	NR	NR
*B*. *cenocepacia* K56-2	81.3 (± 40.0)	96.7 (± 11.5)	NR	51.5 (± 44.1)
*B*. *cenocepacia* C5424	NR	ND	ND	ND
*B*. *cenocepacia* AU1054	97.4 (± 10.4)	ND	98.2 (± 1.4)	ND
*B*. *cenocepacia* LMG18828	75.8 (± 51.1)	NR	ND	ND
*B*. *cenocepacia* LMG18829	95.3 (± 5.7)	69.9 (± 30.5)	NR	97.2 (± 6.2)
*B*. *multivorans* LMG13010^T^	NR	NR	NR	98.1 (± 3.2)
*B*. *multivorans* LMG18825	NR	NR	NR	NR
*B*. *ambifaria* LMG19182^T^	NR	76.7 (± 32.2)	NR	97.0 (± 7.6)

Data shown are percentage reduction in CFU/ml (±SD) when combination treatment is compared to the antibiotic alone (n = 3). NR, no significant reduction in CFU/ml when BH is added to the antibiotic treatment (p > 0.05). ND, not determined because MIC > 1024 μg/ml. Tobramycin (TOB) + BH (TOB+BH), gentamicin (GN) + BH (GN+BH), kanamycin (KN) + BH (KN+BH) and neomycin (NEO) + BH (NEO+BH).

All *B*. *cenocepacia* strains, except *B*. *cenocepacia* C5424, showed an increased susceptibility towards TOB in combination with BH. For GN, KN and NEO, the potentiating effect of BH was strain-dependent. For *B*. *ambifaria* LMG 19182, an increased susceptibility was observed towards GN and NEO in combination with BH. For *B*. *multivorans* strains, the addition of BH only caused an increased susceptibility for *B*. *multivorans* LMG 13010 in combination with NEO (Table 2). The findings for this strain are in contrast with previously obtained data by Brackman et al. [24], where BH did show a TOB-potentiating activity. However, the experimental setup of biofilm formation differs in both studies. Brackman et al. [24] used medical-grade silicone disks placed in 24-well plates, while 96-well microtiter plates were used in this study. These results indicate that the potentiating effect of BH is not only strain- and aminoglycoside-dependent, but also model-system dependent.

For subsequent experiments we used *B*. *cenocepacia* J2315 as the test strain and TOB as the aminoglycoside.

#### Effect of baicalin hydrate on gene expression in B. cenocepacia J2315 biofilms

**Fig 2:**
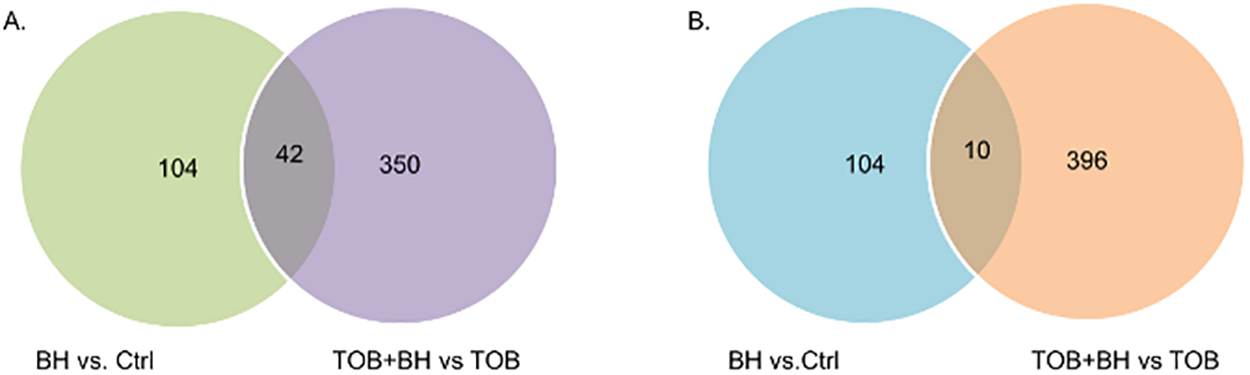
Differentially expressed genes in *B*. *cenocepacia* J2315 biofilms exposed to different treatments. A. Downregulated genes. B. Upregulated genes.

To discover the molecular mechanism by which BH affects biofilm susceptibility towards TOB, transcriptomes of treated and untreated *B*. *cenocepacia* J2315 biofilm cells were compared using RNA sequencing. Results show that the addition of BH had a small but significant impact on gene expression, both for TOB treated and untreated cells (Fig 2). Major differences in gene expression were observed in pathways related to cellular respiration and QS. The genes significantly differentially expressed in these pathways are shown in Table 3. Genes responsible for the electron transport chain and TCA were upregulated, while the expression for genes encoding enzymes of the glyoxylate shunt showed a significant downregulation. These results point to a potential increase in intracellular oxidative stress, as Van Acker et al. [10] previously described an upregulation of glyoxylate shunt-related genes and a downregulation of genes related to the TCA cycle in *B*. *cenocepacia* biofilm cells after treatment with high concentrations of TOB. These cells were likely metabolically less active which leads to reduced ROS production [10]. We hypothesized that BH could stimulate cellular respiration, which subsequently would induce the production of ROS and lead to increased killing. As the oxidative stress response is partially controlled by QS [7] and as BH has already been described as a QS inhibitor [24,35] the focus in the search for the molecular mechanism of BH on the increase of the antibiotic susceptibility of *B*. *cenocepacia* biofilms was directed toward both QS and oxidative stress.

**Table 3:** Differences in gene expression expressed as fold change (p < 0.05) caused by BH (compared to TOB or to an untreated control) in *B*. *cenocepacia* J2315.

Gene number	Annotation	BH vs. ctrl	TOB+BH vs. TOB
**Glyoxylate shunt**			
BCAL2122 (*aceB*)	Malate synthase	-	-1.4
BCAL2118 (*aceA)*	Isocitrate lyase AceA	-	-1.5
BCAM1588	Isocitrate lyase	-	-1.9
**TCA cycle**			
BCAM0972 (*gltA*)	Type II citrate synthase	-	1.8
BCAM0961 (*acnA*)	Aconitate hydratase	-	1.7
BCAL1215 (*lpdV*)	Dihydrolipoamide dehydrogenase	-	1.5
BCAL1517 (*odhL*)	Dihydrolipoamide dehydrogenase	-	1.7
BCAM1250	Putative acetyl-CoA hydrolase/transferase	1.6	1.5
BCAM0970 (*sdhB*)	Succinate dehydrogenase iron-sulfur protein	-	1.6
**Pyruvate metabolism**			
BCAM1581 (*pckG*)	Phosphoenolpyruvate carboxykinase	-	2.0
BCAL1910	Acetoin:2,6-dichlorophenolindophenol oxidoreductase beta subunit	1.6	-
**Oxidative phosphorylation**			
BCAL2337	NADH dehydrogenase I chain H	-	1.5
BCAL2336	NADH dehydrogenase I chain I	-	1.8
BCAL2335 (*nuoJ*)	NADH dehydrogenase I chain J	-	1.5
BCAL2334 (*nuoK*)	NADH-ubiquinone oxidoreductase I chain K	-	1.8
BCAL2333 (*nuoL*)	NADH-ubiquinone oxidoreductase I chain L	-	1.5
BCAL2332 (*nuoM*)	NADH-ubiquinone oxidoreductase I chain M	-	1.6
BCAL2331 (*nuoN*)	NADH dehydrogenase I chain N	-	1.6
BCAM0905 (*ndh*)	Putative NADH dehydrogenase	-	-1.4
BCAM0166 (*ndh*)	NADH dehydrogenase	-2.6	-
BCAM0970 (*sdhB*)	Succinate dehydrogenase iron-sulfur protein	-	1.6
BCAL0759 (*ubiA*)	Prenyltransferase family protein	-	1.4
BCAL2141 (*cyoD*)	Cytochrome O ubiquinol oxidase protein	-	1.6
BCAL0752	Putative cytochrome c oxidase assembly protein	-	1.6
BCAM1734	Putative cytochrome C	-	1.7
BCAL2142 (*cyoC*)	Cytochrome o ubiquinol oxidase subunit III	-	2.0
BCAL2143 (*cyoB*)	Ubiquinol oxidase polypeptide I	-	1.5
BCAM2674	Putative cytochrome oxidase subunit I	-1.6	-
BCAL0784 (*cydB*)	Cytochrome d ubiquinol oxidase subunit II	-	1.5
BCAL0034 (*atpA*)	ATP synthase alpha chain	-	1.7
BCAL0031 (*atpE*)	ATP synthase C chain	-	1.7
BCAL2622 (*ppa*)	Polyphosphate kinase	-	-1.5
**Glucarate/galactarate metabolism to 2-oxo-glutarate**			
BCAL1043 (*gudD*)	Glucarate dehydratase	2.6	1.5
BCAM2511 (*garD*)	Putative galactarate dehydratase	2.3	1.6
BCAM2512	5-dehydro-4-deoxyglucarate dehydratase	2.2	2.9
BCAM2514*	Putative fatty aldehyde dehydrogenase	2.0	1.6
**Quorum sensing**			
BCAM1870 (*cepI*)	N-acylhomoserine lactone synthase CepI	-	1.5
BCAM0239a (*cciI*)	N-acylhomoserine lactone synthase	-	-1.6
BCAM0240 (*cciR*)	N-acylhomoserine lactone dependent regulatory protein	-	-2.3
**Oxidative stress response**			
BCAS0085 (*ohr*)	Organic hydroperoxide resistance protein	-	-1.7
BCAL3477	Putative catalase	-	-1.5
BCAL3301 (*oxyR*)	Oxidative stress regulatory protein	-	-1.8
BCAL2643 (*sodC*)	Superoxide Dismutase SodC	-	-1.5
BCAL2641	Putative ornithine decarboxylase	-	-2.1
BCAM1812	Agmatinase	-1.8	-1.4

#### Effect of baicalin hydrate on oxidative stress

**Fig 3:**
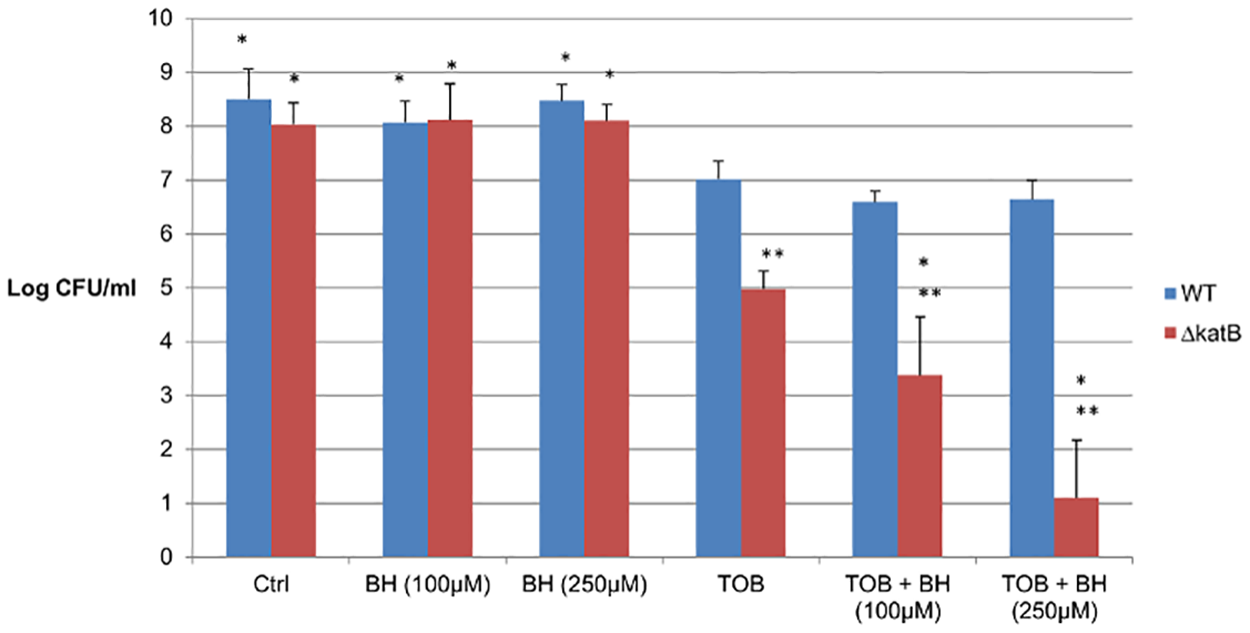
Potentiating effect of BH in *B*. *cenocepacia* Δ*katB*. Data shown are the average log(CFU/ml) recovered after 24h treatment of mature biofilms of *B*. *cenocepacia* C5424 (WT) and its catalase deletion mutant (*ΔkatB*) with 4 x MIC TOB (MIC for both strains = 128 μg/ml), and TOB in combination with BH (100 μM and 250 μM). *: significant difference (p < 0.05) compared to TOB alone **: significant difference (p < 0.05) compared to the wild type. Error bars represents SD (n = 3).

The effect of BH on oxidative stress was evaluated by testing the susceptibility of a catalase deletion mutant (Δ*katB*) and the corresponding wild type strain (*B*. *cenocepacia* C5424) towards the combination BH+TOB. We hypothesised that if BH increases ROS-mediated killing by antibiotics, a mutant that lacks protection against oxidative stress would be more sensitive towards the potentiating effect of BH than the wild type. As shown in Fig 3, there was no increase in susceptibility for the wild type after combining BH with TOB. As previously described [16], TOB treatment of the *katB* deletion mutant resulted in more killing than in the wild type. Furthermore, addition of BH led to a significant further increase in susceptibility of the biofilm cells to TOB in the mutant (but not in the WT). In addition, the effect was more pronounced using higher concentrations of BH, suggesting a dose-dependent effect (Fig 3).

From the transcriptomic analysis we learned that no changes in expression were observed for respiration-related genes upon exposure to BH alone, suggesting the effect of BH on biofilm susceptibility is antibiotic-mediated. This was confirmed by the lack of an effect by BH alone on WT or Δ*katB* biofilms (Fig 3).

**Fig 4:**
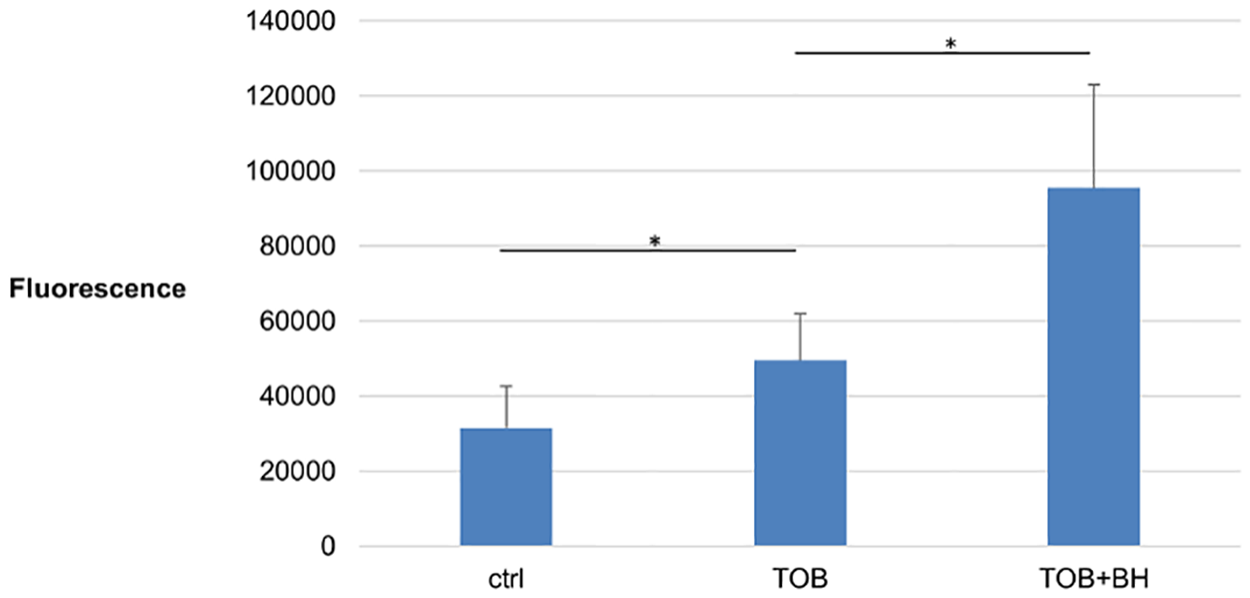
ROS production in *B*. *cenocepacia* J2315 biofilms after treatment with TOB alone or in combination with BH. Accumulation of ROS in *B*. *cenocepacia* J2315 biofilms, expressed as fluorescence generated after incubation with H_2_DCFDA, after 24 hours treatment with TOB (4 x MIC), TOB in combination with BH (250 μM) or an untreated pH-matched control. Data presented are means, error bars are standard deviations. The experiment was conducted six times. *: Significant difference (p < 0.05) compared to treatment with TOB alone.

To confirm the role of BH in promoting ROS-mediated killing, endogenous ROS accumulation was measured using the H_2_DCFDA assay. In this assay, fluorescence generated is a measure for the amount of ROS present in the cell. Almost a 2-fold increase in fluorescence is observed when *B*. *cenocepacia* J2315 biofilms were treated with TOB compared to the untreated control. Another 2-fold increase is observed when BH is combined with TOB, compared to TOB alone (Fig 4). This confirms an increased production of ROS in the cells treated with TOB+BH.

#### Baicalin hydrate as a quorum sensing inhibitor

**Fig 5:**
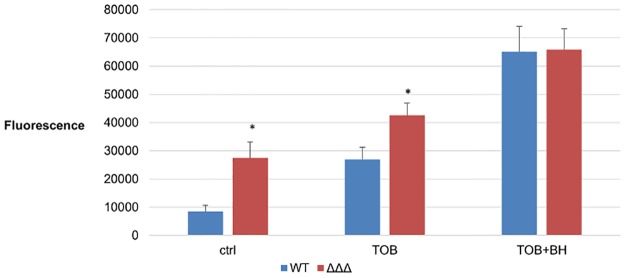
ROS production in *B*. *cenocepacia* J2315 and its triple QS mutant after treatment with TOB alone or in combination with BH. Accumulation of ROS, expressed as fluorescence (average ± SD) generated after incubation with H_2_DCFDA, in planktonic cultures of *B*. *cenocepacia* J2315 and its triple QS mutant treated with TOB (4 x MIC) or the combination with BH (250 μM) and a pH-matching control after 16 hours. MIC for TOB was 256 μg/ml and 128 μg/ml for the wild type and its triple QS mutant respectively. The experiment was conducted using six biological replicates. *: statistically significant difference compared to the wild type (p < 0.05).

As the oxidative stress response is co-regulated by QS [7,16] and as BH has been described as a QS inhibitor [24], we hypothesized that BH inhibits QS and as a result increases ROS production in *B*. *cenocepacia*. To test this hypothesis, ROS production in a triple QS mutant (Δ*cepI*Δ*cciI*Δ*rpfF*) was compared to ROS production in the wild type after treatment with TOB and BH. A triple QS mutant was chosen over single Δ*cepI* or Δ*cciI* mutants in order to avoid biased results caused by the complex interaction between the three QS networks in *B*. *cenocepacia* J2315 [26,36]. The H_2_DCFDA assay was carried out on planktonic cells to eliminate nonspecific effects due to the reduced biofilm formation of the triple QS mutant [26]. We observed a significant increase in the amount of ROS in the triple QS mutant compared to the wild type for the control and TOB treatments (Fig 5). The increased amount of ROS is probably due to a lack of oxidative stress response in the triple QS mutant, as previously described [16]. Surprisingly, the addition of BH resulted in an increased ROS production in the triple QS mutant compared to TOB alone. Also, no difference was observed between the triple QS mutant and the wild type for the combination treatment. Whether these findings mean that the effect of BH on oxidative stress is unrelated to QS or whether the maximal amount of ROS has been reached (and cannot further be increased by addition of BH) remains to be determined.

Remarkably, the data obtained from RNA sequencing revealed an upregulation of genes involved in the main QS systems when BH and TOB were combined (Table 3). *cciR* (BCAM0240) and *cciI* (BCAM0239A) are both located on chromosome 2, and are co-transcribed [37]. They encode CciR and CciI, which are mainly negative regulators of QS-related genes, and showed a significant downregulation of 2.3 and 1.6 fold, respectively. *cepI* (BCAM1870) and *cepR* (BCAM1868) are also located on chromosome 2, but are divergently transcribed [37]. CepI, the synthase of the CepIR system which is mainly a positive regulator, was 1.5 fold upregulated. These results are in accordance with results from a previous study in which an upregulation of *cepI* was observed in several stress conditions (including low oxygen and high temperature) [13]. It is conceivable that the upregulation of these QS systems is not a direct result of the presence of BH, but rather an indirect effect, possibly due to differences in growth stages after both treatments. This is in agreement with observations by Brackman et al [38], where the addition of BH to biofilms at the same growth stage resulted in a downregulation in expression of QS-regulated genes.

Based on these results, we could not confirm a direct link between QS and the effect of BH on oxidative stress. Therefore other mechanisms were considered in the search of a mode of action for BH.

#### Influence of baicalin hydrate on cellular respiration

An upregulation of the expression of genes involving the oxidative phosphorylation and TCA cycle was observed upon the addition of BH to TOB treatment (Table 3). This suggests that BH increases respiration, which could increase TOB-mediated killing.

**Fig 6:**
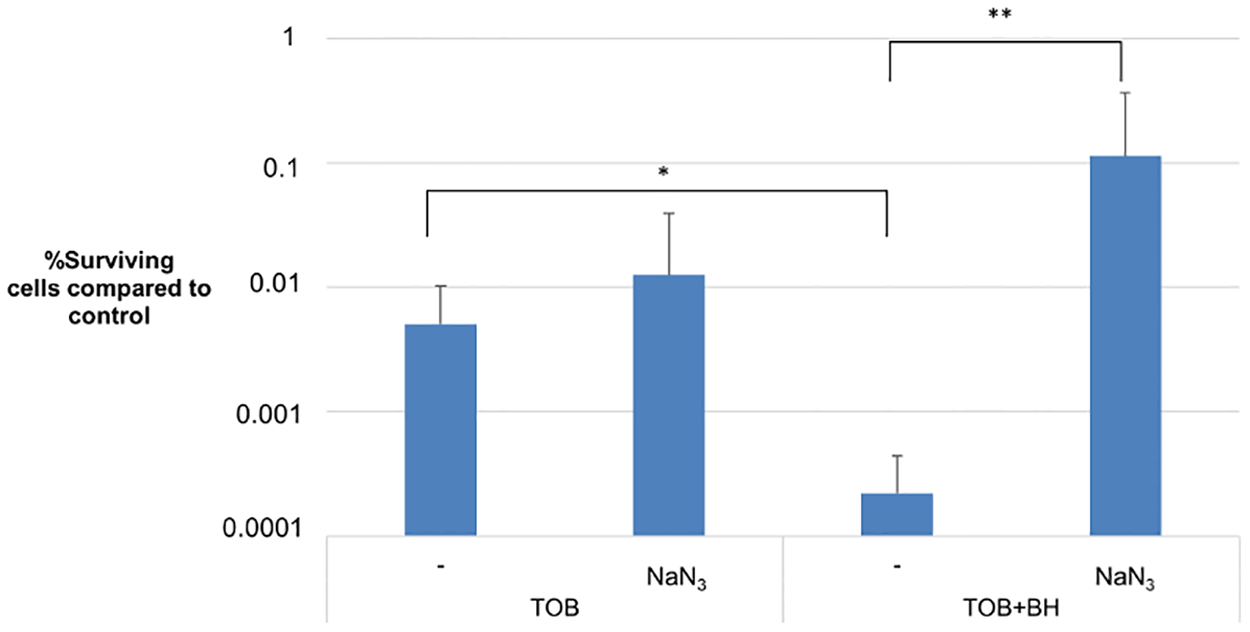
Effect of electron transport chain inhibition by NaN_3_ on BH-mediated TOB potentiation. Percentage of surviving cells (±SD) after treatment compared to their respective controls (which received a pre-treatment but no antibiotic). Final concentrations of NaN_3_, BH and TOB were 150 μM, 250 μM and 1024 μg/ml (4 x MIC) respectively. Pre-treated cells received BH, NaN_3_, a combination of both or MHB for 4 hours. The experiment was conducted in triplicate. *: significant difference (p < 0.05) between sessile cells not treated with NaN_3_. **: significant difference (p < 0.05) between sessile cells when NaN_3_ is included in the treatment.

To evaluate the influence of BH on oxidative phosphorylation, the effect of a cytochrome c oxidase inhibitor (sodium azide, NaN_3_) on the potentiation of TOB by BH was investigated. Biofilms were pre-treated with NaN_3_, BH, or a combination of both. After 4 hours, TOB was added to the pre-treated cells for an additional 20 hours. Data in Fig 6 depict the percentage of surviving cells compared to their respective controls. There is no increase in surviving cells between sessile cells treated with TOB and NaN_3_ compared to TOB alone. However, when NaN_3_ was combined with TOB+BH, a significant increase in surviving cells could be observed compared to TOB+BH alone, showing that the addition of NaN_3_ suppressed the potentiating effect of BH. These results are in accordance with data showing increased production of ROS (Fig 4), since an increased activity of the electron transport chain will result in an increased production of ROS [11]. Together our data suggest an influence of BH on the proton motive force, leading to potentiation of the activity of TOB. It was previously shown that metabolic stimulation of the TCA cycle can increase susceptibility towards aminoglycosides, but not to other classes of antibiotics [39]. This is in line with the findings in the present study.

#### Influence of baicalin hydrate on glucarate metabolism

As RNAseq data revealed an upregulation of genes involved in cellular respiration, we looked for changes in the expression of genes involved in turnover of compounds feeding into the TCA cycle.

**Fig 7:**
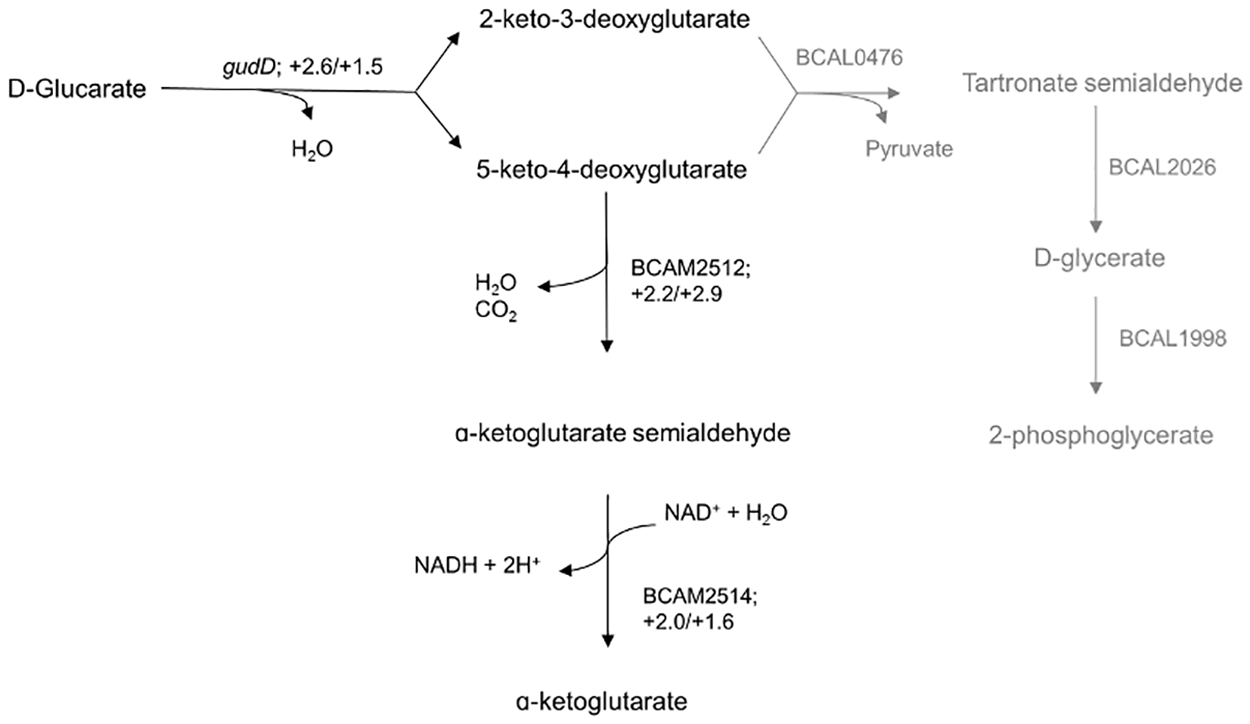
BH affects regulation of genes involved in glucarate metabolism of *B*. *cenocepacia* J2315. The reactions depicted in black are significantly (p < 0.05) upregulated (fold changes of “BH vs. Ctrl” / “TOB+BH vs TOB”). The involved enzymes are *gudD* (glucarate dehydratase), BCAM2512 (5-keto-4-deoxyglutarate dehydratase) and BCAM2514 (ɑ-ketoglutarate semialdehyde dehydrogenase). For the reactions depicted in grey no significant differential expression in either “BH vs. Ctrl” or “TOB+BH vs TOB” was observed.

The only pathway with a direct link to the TCA cycle that showed upregulation of multiple genes was that for glucarate utilisation (Fig 7). D-glucarate, the dicarboxylic acid analogue of glucose, can serve as a growth substrate in many bacteria [40]. According to the biochemical pathways in the KEGG database, *B*. *cenocepacia* J2315 is able to use two pathways for the utilization of D-glucarate [31]. In the first pathway D-glucarate is converted to D-glycerate and finally to 2-phosphoglycerate, which is a metabolite in the glycolytic pathway [41]. In the second pathway three enzymatic steps lead to the generation of ɑ-ketoglutarate as an end product, which is a key substrate in the TCA cycle [42].

The expression of genes involved in the pathway generating D-glycerate was unaffected by addition of BH. However, in the other pathway, a significant upregulation (2.6-fold) was observed for glucarate dehydratase (*gudD*) upon the addition of BH. Also for 5-keto-4-deoxyglutarate dehydratase (BCAM2512) and ɑ-ketoglutarate semialdehyde dehydrogenase (BCAM2514), genes coding for enzymes involved in generating ɑ-ketoglutarate [43], a significant upregulation could be observed (2.2-fold and 2.0-fold, respectively). This was also the case when gene expression was compared between cells exposed to the combination of TOB and BH, and those exposed to TOB alone: *gudD*, BCAM2512 and BCAM2514 showed a 1.5-fold, 2.9-fold, and 1.6-fold increased expression, respectively (Fig 7).

**Fig 8:**
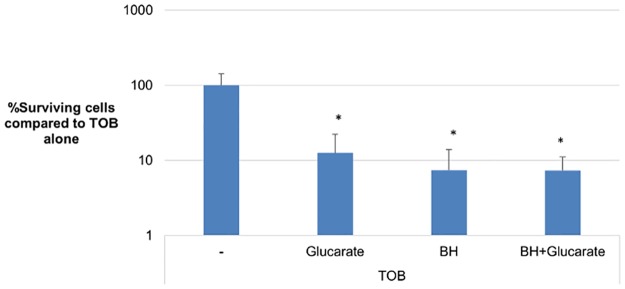
Impact of glucarate and BH on the susceptibility of *B*. *cenocepacia* J2315 biofilms towards TOB. Data shown are the percentage surviving cells compared to TOB treatment alone. *: significantly less surviving cells compared to TOB alone (p < 0.05). Error bars show SD (n = 3).

To further investigate the involvement of glucarate metabolism in the potentiating activity of BH, glucarate was added to sessile cells treated with TOB and TOB+BH. The glucarate+TOB treatment caused a significant reduction in the number of surviving cells compared to TOB alone. This reduction was similar to that observed for the combinations TOB+BH and TOB+BH+glucarate (Fig 8). Our data suggest that stimulation of the glucarate degradation pathway (by adding glucarate or BH) increases cellular metabolism and increases susceptibility to TOB. When both compounds are added simultaneously, the pathway is not more stimulated than it is by either one of the compounds, resulting in a similar reduction in surviving cells.

#### Influence of baicalin hydrate on putrescine biosynthesis

**Fig 9:**
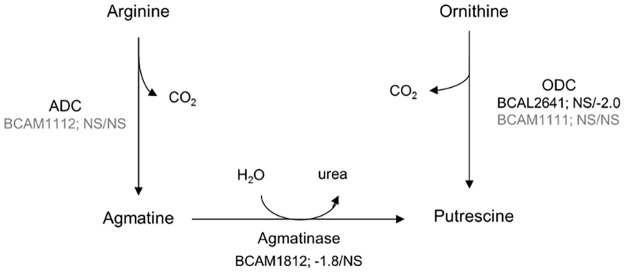
BH affects regulation of genes involved in putrescine biosynthesis of *B*. *cenocepacia* J2315. The reactions depicted in black are significantly (p < 0.05) differentially regulated (fold changes of “BH vs. Ctrl” / “TOB+BH vs TOB”). The enzymes involved the putrescine synthesis pathway are ornithine decarboxylase (ODC), arginine decarboxylase (ADC) and agmatinase. NS: no significant change in gene expression (p > 0.05).

Bacteria can produce polyamines that quench ROS and protect membranes against lipid peroxidation [11]. Polyamines are small aliphatic molecules with multiple amino groups, which are protonated at physiological pH. The most common cellular polyamines are putrescine, spermidine, spermine and cadaverine [44]. The most abundant one in *B*. *cenocepacia* is putrescine, whereas spermidine and cadaverine are produced in lower amounts [45]. *B*. *cenocepacia* can produce putrescine via two different pathways. In the first pathway ornithine decarboxylase (ODC) converts ornithine to putrescine. The second pathway uses arginine as a start product, which is decarboxylated to agmatine by arginine decarboxylase (ADC). In a following step, agmatine is converted to putrescine by agmatinase, releasing urea [28]. *B*. *cenocepacia* has two ODC homologues (BCAM1111 and BCAL2641) and one ADC homologue (BCAM1112) (Fig 9). El-Halfawy et al. [28] demonstrated that these three genes are the only contributors to putrescine production in *B*. *cenocepacia*, and that BCAL2641 is the key enzyme in protection against antibiotic-mediated oxidative stress. They also showed that the ODC BCAL2641 responds to antibiotic stress by increasing putrescine levels. The other ODC BCAM1111 and ADC BCAM1112 were not affected by exogenous stress and their expression appeared to be regulated by BCAL2641. This suggests that the increased levels of putrescine upon antibiotic stress depend on the activity of BCAL2641. Increased putrescine levels can induce expression of *oxyR* which activates oxidative stress response mechanisms, whereas a reduced putrescine biosynthesis resulted in an increased ROS generation [28].

**Fig 10:**
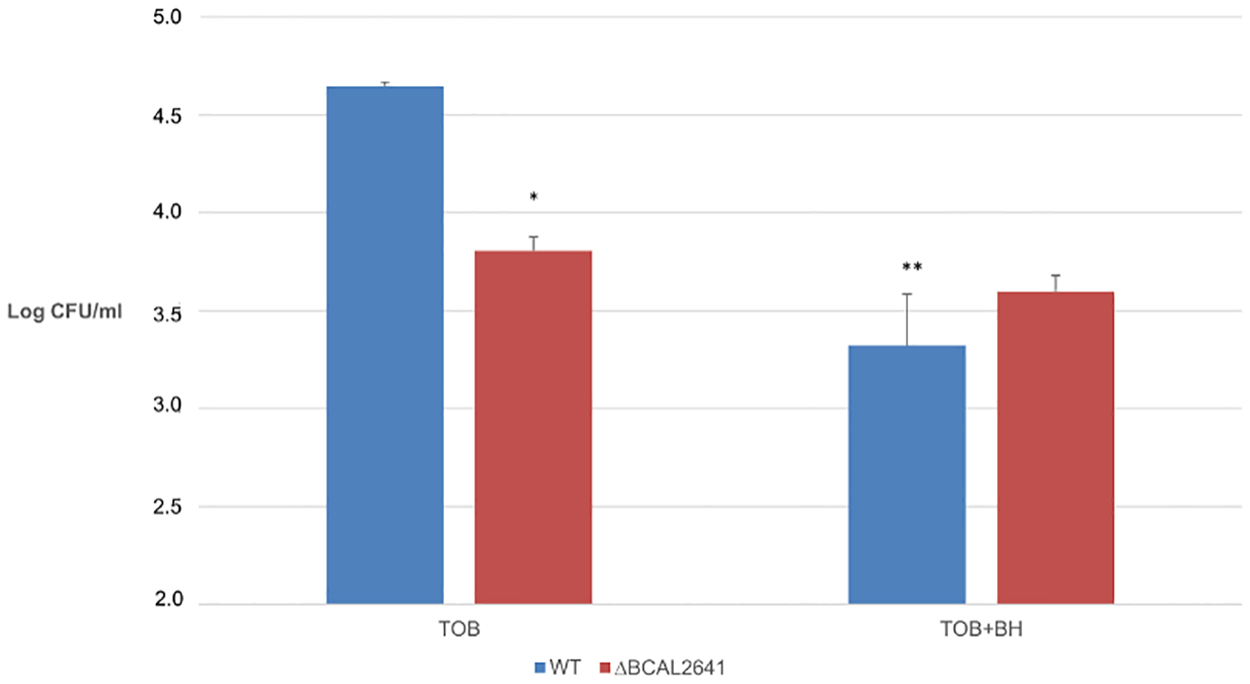
Influence of BH on TOB susceptibility of *B*. *cenocepacia* K56-2 biofilms and its ΔBCAL2641 mutant. Data are averages of log(CFU/ml) surviving cells after treatment with TOB (8 x MIC) alone or in combination with BH (250 μM). Influence of BH on biofilm susceptibility was evaluated in *B*. *cenocepacia* K56-2 (wild type) and its ΔBCAL2641 deletion mutant. The MIC for TOB in both strains was 128 μg/ml. The experiment was conducted in triplicate. *: significantly different compared to the wild type (p < 0.05). **: significant difference compared to TOB alone (p < 0.05). Error bars are SD.

The key enzyme in putrescine biosynthesis (ODC, BCAL2641) was significantly downregulated (-2.1-fold) in cells treated with TOB+BH compared to treatment with TOB alone. Since this enzyme protects against oxidative stress in *B*. *cenocepacia* [28], we hypothesized that BH causes a downregulation of BCAL2641 which would lead to an inhibition of putrescine synthesis, resulting in impaired oxidative stress response leading to increased biofilm susceptibility towards TOB. To test this hypothesis, we investigated the potentiating effect of BH in a ΔBCAL2641 mutant and the corresponding WT strain (*B*. *cenocepacia* K56-2) [28]. Biofilms were treated with TOB alone (8 x MIC) and a combination of TOB (8 x MIC) and BH (250 μM) (Fig 10). The ΔBCAL2641 deletion mutant is more susceptible to TOB than the wild type, indicating that putrescine protects against oxidative stress, as previously described [12,28]. Furthermore, there is no difference between wild type and mutant when cells were treated with TOB + BH. This could indicate that the potentiating effect of BH is indeed linked to regulation of BCAL2641 expression by BH. Together, these results suggest that BH affects putrescine biosynthesis, and by doing so affects the oxidative stress response, leading to an increased biofilm susceptibility.

## Concluding remarks

Several studies already indicated that changes in metabolism upon antibiotic treatment play an important role in the effect of antibiotics [10,15,19,20,22]. These metabolic shifts allow the bacteria to enter a protective state by reducing cellular growth, by limiting ROS production [46] and/or by reducing antibiotic uptake [39]. Especially aminoglycosides can be affected by the latter, since their uptake is an energy-requiring process [39].

In conclusion, the addition of BH to TOB treatment increases oxidative stress in *B*. *cenocepacia* J2315 biofilms compared to treatment with TOB alone. The potentiating activity of BH appears to be strain-, aminoglycoside- and model-system dependent. While the exact mode of action is still not entirely clear, we have shown that BH has an impact on oxidative stress by influencing oxidative phosphorylation, glucarate metabolism and the protective response by putrescin. Combined, these factors cause an increased ROS production and increased killing upon exposure to TOB.

## Supporting information

S1 Fig Potentiating effect of BH in combination with several antibiotics on *B*. *cenocepacia* J2315 biofilms. Data shown are percentage survival of *B*. *cenocepacia* J2315 biofilm cells treated with the combination of BH (250 μM) with antibiotic compared to the antibiotic alone (4 x MIC) (MICs are shown in S1 Table). The antibiotics are ceftazidime (CEF), ciprofloxacin (CIP), minocycline (MIN), meropenem (MEM) and co-trimoxazole (SXT). None of the combination treatments were significantly different (p > 0.05) compared to the antibiotic alone (n = 3).
(TIF)

S1 Table MICs of *B*. *cenocepacia* J2315 for several antimicrobial agents. (XLSX)

S2 Table MIC (μg/ml) of other tested Bcc species for several aminoglycosides, including tobramycin (TOB), kanamycin (KN), neomycin (NEO) and gentamicin (GN). (XLSX)
