# Supplementary figures and images for: Elucidation of the mechanism behind the potentiating activity of baicalin against Burkholderia cenocepacia biofilms
Source: PLoS One. 2018 Jan 2;13(1):e0190533. doi: 10.1371/journal.pone.0190533 (PMC5749847; doi:10.1371/journal.pone.0190533)

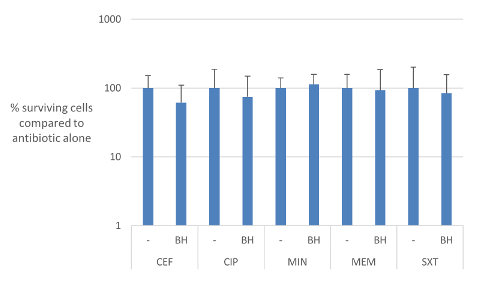

Supplement: S1 Fig — Data shown are percentage survival of B. cenocepacia J2315 biofilm cells treated with the combination of BH (250 μM) with antibiotic compared to the antibiotic alone (4 x MIC) (MICs are shown in S1 Table). The antibiotics are ceftazidime (CEF), ciprofloxacin (CIP), minocycline (MIN), meropenem (MEM) and co-trimoxazole (SXT). None of the combination treatments were significantly different (p > 0.05) compared to the antibiotic alone (n = 3). (TIF) [file pone.0190533.s001.tif]
